# Persistent neurological symptoms and elevated intracranial pressures in a previously healthy host with cryptococcal meningitis

**DOI:** 10.1186/s12879-023-08349-y

**Published:** 2023-06-14

**Authors:** Mohammad El-Atoum, Jessica C. Hargarten, Yoon-Dong Park, Kenneth Ssebambulidde, Li Ding, Prashant Chittiboina, Dima A. Hammoud, Seher H. Anjum, Seth R. Glassman, Shehzad Merchant, Peter R. Williamson, John C. Hu

**Affiliations:** 1 Department of Medicine, Good Samaritan Hospital, SSM Health Medical Group, Mount Vernon, IL USA; 2 Laboratory of Clinical Immunology and Microbiology, National Institute of Allergy and Infectious Diseases, NIH, Bethesda, MD USA; 3 Surgical Neurology Branch, National Institute of Neurological Disorders and Stroke, National Institutes of Health, Bethesda, MD USA; 4 Center for Infectious Disease Imaging (CIDI), Radiology and Imaging Sciences, Clinical Center, National Institutes of Health, Bethesda, MD USA; 5 Department of Medicine, Division of Infectious Diseases, University at Buffalo, 955 Main Street, Buffalo, NY 14203 USA

**Keywords:** Cryptococcus, Intracranial hypertension, Neuro-inflammation, Corticosteroids, Post infectious inflammatory response syndrome

## Abstract

Cryptococcal meningoencephalitis can occur in both previously healthy and immunocompromised hosts. Here, we describe a 55 year-old HIV-negative male with no known prior medical problems, who presented with three months of worsening headaches, confusion, and memory changes without fever. Magnetic resonance imaging of the brain demonstrated bilateral enlargement/enhancement of the choroid plexi, with hydrocephalus, temporal and occipital horn entrapments, as well as marked periventricular transependymal cerebrospinal fluid (CSF) seepage. CSF analysis yielded a lymphocytic pleocytosis and cryptococcal antigen titer of 1:160 but sterile fungal cultures. Despite standard antifungal therapy and CSF drainage, the patient had worsening confusion and persistently elevated intracranial pressures. External ventricular drainage led to improved mental status but only with valve settings at negative values. Ventriculoperitoneal shunt placement could thus not be considered due to a requirement for drainage into the positive pressure venous system. Due to this persistent CSF inflammation and cerebral circulation obstruction, the patient required transfer to the National Institute of Health. He was treated for cryptococcal post-infectious inflammatory response syndrome with pulse-taper corticosteroid therapy, with resultant reductions in CSF pressures along with decreased protein and obstructive material, allowing successful shunt placement. After tapering of corticosteroids, the patient recovered without sequelae. This case highlights (1) the necessity to consider cryptococcal meningitis as a rare cause of neurological deterioration in the absence of fever even in apparently immunocompetent individuals and (2) the potential for obstructive phenomena from inflammatory sequelae and the prompt response to corticosteroid therapy.

## Introduction

Cryptococcosis is an infectious disease with worldwide distribution and a wide array of clinical presentations caused by encapsulated yeasts in the genus *Cryptococcus*. Currently, two species of *Cryptococcus* commonly cause disease in humans: *Cryptococcus neoformans* and *Cryptococcus gattii.* [1, 2] *Cryptococcus* species can cause fatal meningoencephalitis in immunocompromised individuals (e.g. HIV, organ transplant or other immunosuppressive conditions) as well as previously healthy individuals [3]. Although the incidence of cryptococcal meningoencephalitis (CM) has declined significantly since the introduction of anti-retroviral therapy (ART), it remains one of the leading causes of HIV-related deaths in parts of the world [4]. In a study on the epidemiology of CM in the US between 1997 and 2009, the rates of infection have been persistently elevated in non-HIV infected patients with sustained mortality rates despite therapy [5, 6]. In a US study that included more than 300 patients with cryptococcosis, 30% of patients with CNS cryptococcosis had no apparent underlying medical conditions [7].

An important complication in the treatment of CM is attributed to the immune response after effective anti-fungal therapy. In AIDS-related CM, immune reconstitution inflammatory syndrome (IRIS) after initiation of ART is a significant cause of morbidity and mortality [8, 9]. Similar to IRIS, in otherwise previously healthy patients with CM, Post Infectious Inflammatory Response Syndrome (PIIRS) can result in clinical deterioration in the face of effective anti-fungal therapy [10, 11]. Indeed, the immune response within the CNS and its related complications present significant clinical and therapeutic challenges in the management of previously healthy individuals with CM.

Herein, we present a case of cryptococcal meningoencephalitis in an apparently healthy adult with a complicated course due to profound CNS inflammation resulting in the production of substantial inflammatory debris preventing optimal ventricular diversion. This case report aims to emphasize the importance of recognizing cryptococcal infections in immunocompetent patients and to highlight challenges in the management of patients who experience neurological deterioration despite adequate antifungal therapy and standard approaches to CSF drainage.

## Case presentation

**Fig. 1:**
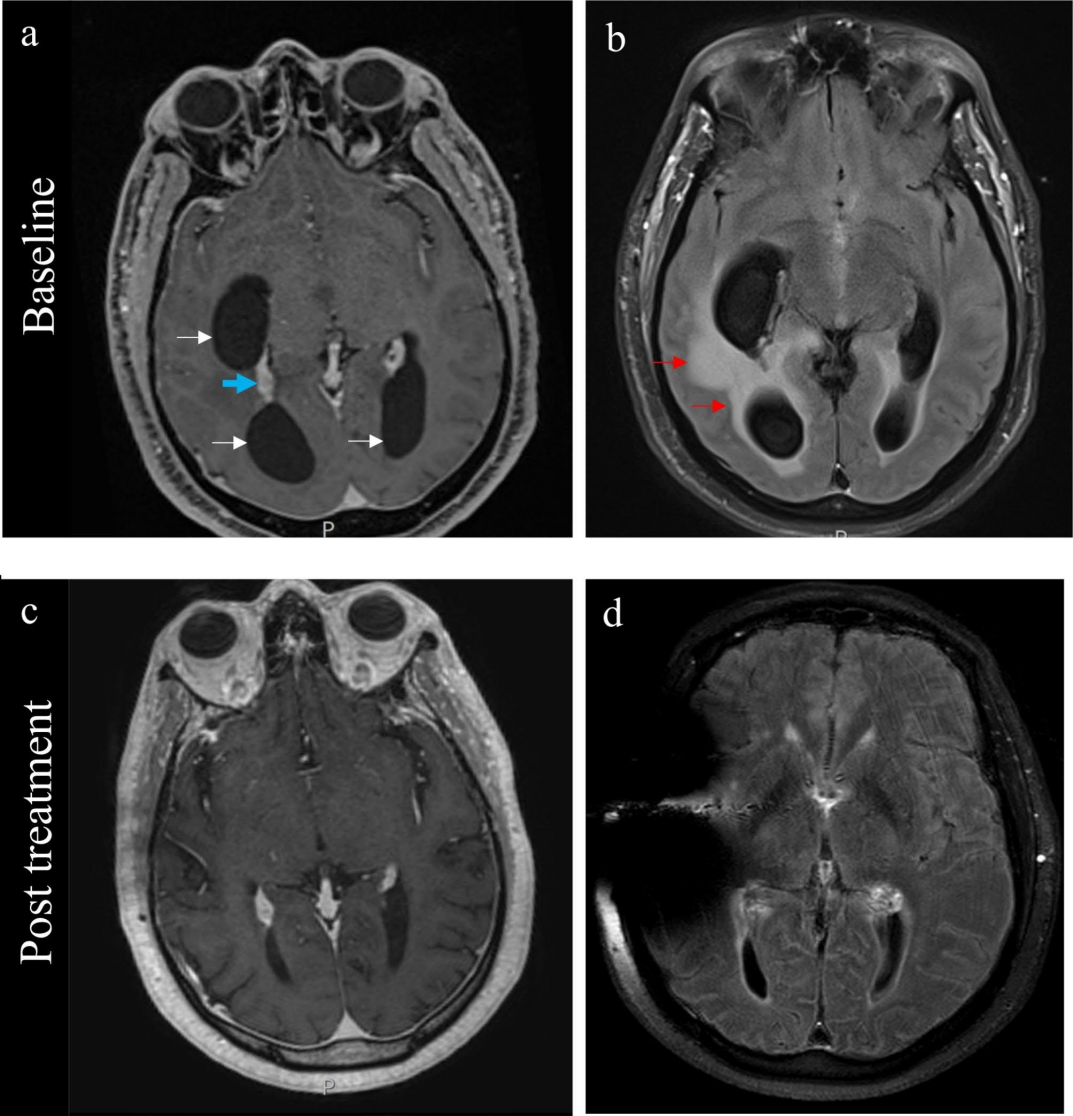
MRI scans of the brain before and after corticosteroid treatment: (**a**) Post-contrast T1-weighted imaging showing significant ventricular enlargement (white arrows) with enhancing choroid plexi (blue arrow) consistent with choroid plexitis. The occipital and temporal horns of the lateral ventricles are entrapped likely due to inflammatory adhesions. (**b**) Post-contrast FLAIR imaging showing periventricular edema consistent with transependymal CSF seepage associated with the enlarged and entrapped ventricles (red arrows). (**c**) Both choroid plexitis and entrapment significantly improved 60 days post steroid treatment with decreased periventricular transependymal CSF seepage (**d**)

A 55-year-old male who worked as a home inspector and no known prior medical problems presented with three months of progressive headaches and confusion. The patient’s symptoms accelerated for two weeks and on the day of admission, was found confused and wandering in the streets. On physical examination he was afebrile, alert but disoriented, and without noted motor or sensory deficits. Laboratory evaluations included an initial white blood cell count (WBC) of 12,600 cell/mm^3^ (4,500 to 11,000 cell/mm^3^) and normal renal and liver functions. Non-contrast CT scan of the brain showed ventriculomegaly and MRI of the brain demonstrated diffuse enhancement and enlargement of the choroid plexi bilaterally with hydrocephalus and superimposed entrapment of the temporal and occipital horns of the lateral ventricle with associated transependymal CSF seepage in the periventricular regions (Fig. 1). The patient was intubated and bilateral external ventricular devices (EVDs) were placed in frontal horns of bilateral lateral ventricles. CSF opening pressure was not recorded during EVD placement but operative note reported very high intracranial pressure. CSF analysis showed pleocytosis with 54 leukocytes per mm^3^ and a differential of 2% neutrophils, 88% lymphocytes, and 10% monocytes. CSF chemistries demonstrated a glucose of 25 mg/dL, lactate of 6.6 (mmol/L), and total protein 374 mg/dl. CSF gram stain and India ink staining were negative and CSF fungal and bacterial cultures were negative. Cryptococcal Ag returned positive at a titer of 1:160. Subsequent polymerase chain amplification of material from the CSF by a previously described method [12] followed by sequencing of the *URA5* gene identified the *Cryptococcus* as a *C. neoformans* var *grubii* (VNII). Serial lumbar punctures were required with results notable for markedly elevated pressures and protein levels (Table 1). Fungal cultures of the CSF were sterile throughout the admission.

**Table 1:** Serial CSF findings during admission. *(n/a = not available, lymp = lymphocytes)*

Admission Day	0	8	14	16	17	24	29	37 NIH Admit	39 Day 2 Pulse Steroids	49 End of Pulse Steroid	51 Day 2 Post Pulse
WBC cell/mm^3^	54	9	1	14	13	140	40	48	88	66	11
Neutrophils (%)	2	2	6	28	3	5	14	n/a	3	n/a	52
Lymphocytes (%)	88	97	77	40	83	74	53	92	80	94	31
Monocytes (%)	10	n/a	17	32	14	20	33	n/a	n/a	n/a	n/a
RBC cell/mm^3^	2,080	710	48	7,450	4,260	23,100	195	12	n/a	n/a	15,750
Protein mg/dl	374	235	377	454	509	438	341	347	312	142	104
Glucose mg/dl	25	45	59	56	60	49	65	54	81	66	60
Cryptococcal Ag titer	1:160	1:160	1:160	1:160	1:160	1:160	n/a	n/a	n/a	n/a	n/a

Evaluation for immunodeficiencies included a negative HIV-1/2 antibody/antigen screen, undetectable HIV RNA viral load, CD4 + T cells of 700 cell/mm^3^ (500-1,200), CD4/CD8 ratio > 1, normal *S. pneumoniae* and tetanus toxoid Ab titers, and quantitative immunoglobulins. Auto-antibodies against GM-CSF were found though determined to be nonfunctional by previously described methods [13]. Other autoantibodies, including those against IFNγ, where negative.

### Anti-fungal treatment

Treatment was initiated with liposomal amphotericin B (L-AMB) 5 mg/kg and 5 flucytosine (5FC) 25 mg every 6 h. Five days after starting therapy, serum creatinine increased to 2.07 from a baseline of 0.87. Due to renal injury, L-AMB was changed to fluconazole 800 mg daily with continuation of 5FC resulting in recovery of renal function. Multiple weaning trials of the EVDs were attempted but failed due to persistent hydrocephalus and bilateral occipital and temporal horn entrapment on repeat MRI brain. Due to progressive mental status deterioration, this patient was restarted on L-AMB and 5FC. Failure of EVD weaning trials were attributed to neuro-inflammation and elevated protein in the spinal fluid possibly causing obstruction of the drain. Endoscopic third ventriculostomy (ETV) was attempted but the procedure aborted given extensive inflammation and scarring in the ventricles. The patient was arousable only after placing the EVD at negative pressures by placing the pressure monitor on the patient room’s floor, which also prevented shunt internalization. The patient was then transferred to the NIH for concerns of a post-infectious inflammatory response syndrome (PIIRS) and management of persistently elevated ICP.

**Fig. 2:**
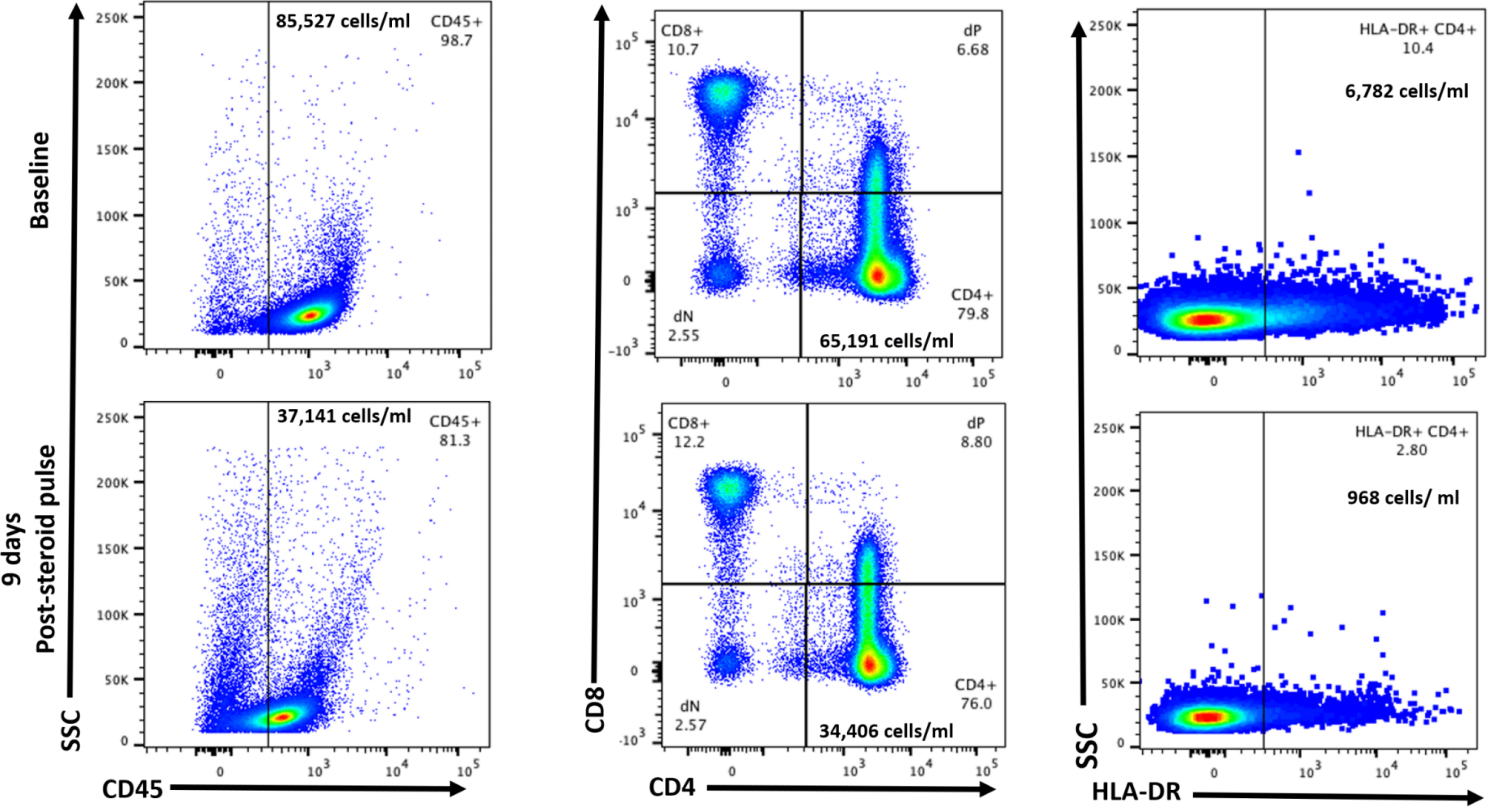
Cerebrospinal fluid flow cytometry showing increased activated HLA-DR + CD4 + T cells at baseline that decreased 9 days after starting steroids

At the NIH, serial CSF fluid analysis demonstrated persistently elevated protein and pleocytosis (Table 1). Additional CSF studies demonstrated increased CD4^+^HLA-DR^+^ cells (6,782 cells/mL, Fig. 2) and elevated levels of IL-13 to 29.4 pg/mL (12-fold), IL-6 to 24 pg/mL (12 fold), IL-8 to 188.7 pg/mL (63 fold), and IL-10 to 11.3 pg/mL (4 fold) and negative fungal cultures. The combination of negative CSF fungal cultures and elevated inflammatory profile associated with clinical decline supported the diagnosis of PIIRS.

**Fig. 3:**
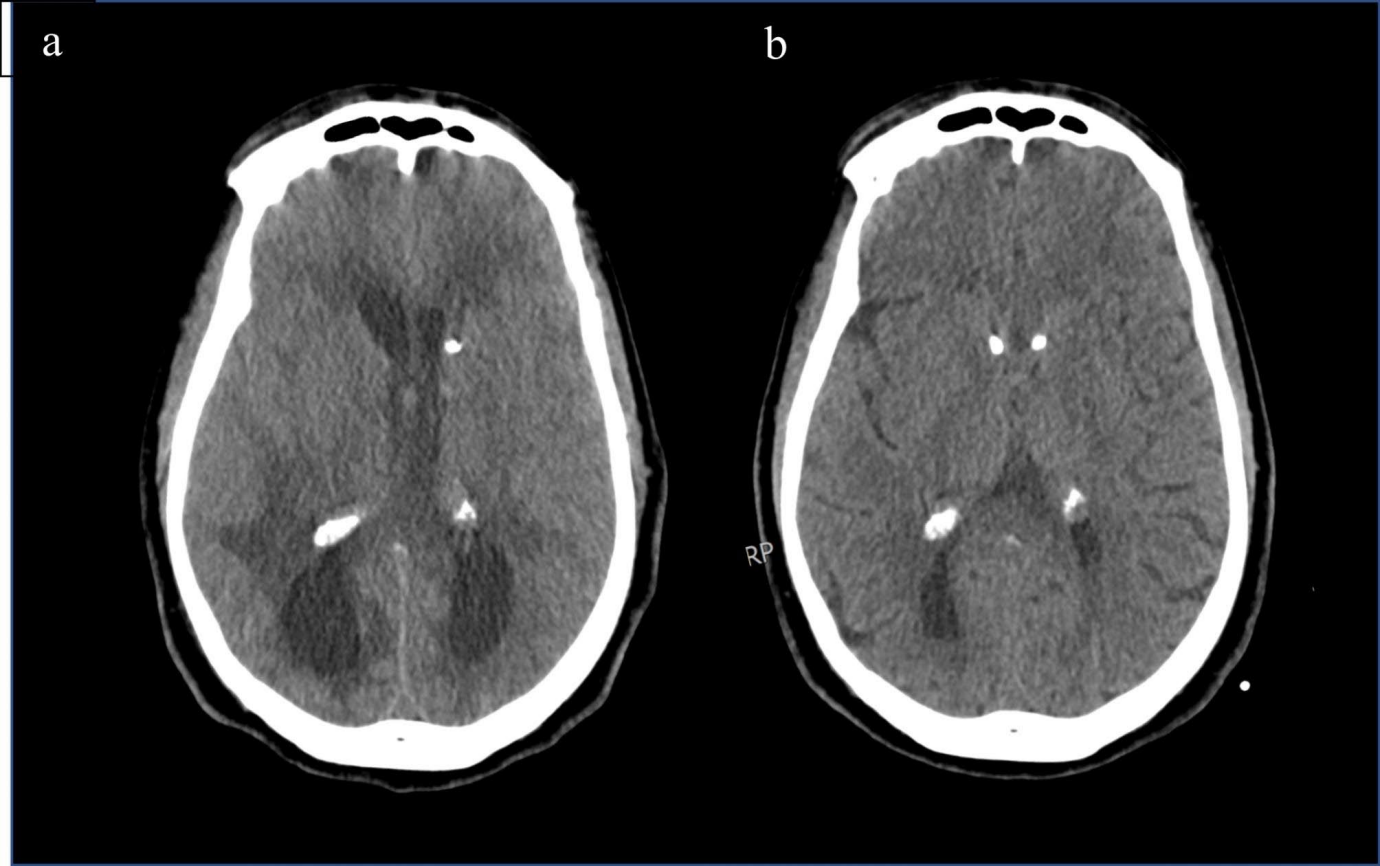
Non-contrast CT of the head after EVD obstruction demonstrating hydrocephalus and periventricular transependymal CSF seepage associated with sulcal effacement (**a**), with resolution of sulcal effacement after internalization of bilateral ventricular-peritoneal shunt drainage (**b**)

For treatment of PIIRS, he was started on methylprednisolone 1 gm IV daily for 7 days with transition to oral prednisone 1 mg/kg/day (60 kg). He completed a 5-day course of L-Amb after NIH transfer and was transitioned to fluconazole 400 mg PO daily because of the negative CSF cultures. However, because of residual CSF inflammatory material, the right EVD acutely obstructed resulting in acute hyponatremia from SIADH/salt wasting syndrome, sulcal effacement on CT head and an acute decline in mental status (Fig. 3a), responding first to a 3% saline drip and then one bolus of 23% hypertonic saline and emergent replacement of the EVD. Subsequent to this, CSF total protein and inflammatory material declined over subsequent days (Table 1), resulting in no further obstructions. Twenty days after initiation of pulse corticosteroids, EVDs were removed and a bifrontal VP shunt was placed for persistent hydrocephalus with significant improvement in bilateral temporal and occipital horn entrapments (Fig. 3b).

A bone marrow biopsy was also performed at the NIH to investigate the cause for anemia and thrombocytopenia which demonstrated a hypocellular marrow with progressive trilineage hematopoiesis and an adequate number of megakaryocytes. There were no overt morphologic features of dysplasia or other primary myeloid disorders. Cytomegalovirus (CMV) was detected in the bone marrow aspirate (4.08 log) and serum via PCR. Further investigation included a negative ophthalmology exam for retinitis and CT chest was unremarkable for pneumonitis but in the setting of anticipated prolonged steroid use and unknown possible genetic immune deficits, the patient was treated with oral valganciclovir until serum CMV levels were negative.

He was discharged home on 80 mg prednisone daily with plans to taper as well as oral fluconazole 400 mg daily. On follow-up visits, he had no recurrence of serious ventricular entrapment on his brain MRI and prednisone was tapered by 5 mg every month over 12 months. Currently, he is off prednisone, valgancyclovir and fluconazole, ambulating without difficulty and without neurological deficit, with return of his previous activities of daily living including his previous work assignments.

### Discussion and literature review

In the US, about 1,000 cases of CM have been reported per year in otherwise healthy hosts [14] with mortality in this patient population as high as 30% [3]. Clinically, CNS cryptococcal infection has a wide range of signs and symptoms, including headaches, fevers, cranial nerve involvement, changes in mental status, memory changes [1] which may last over several weeks to months [1, 15]. As opposed to patients with a known immunodeficiency, immunocompetent individuals with CM usually present in a more indolent fashion and have overwhelming neuro-inflammatory responses which may explain the high mortality rate [15]. Fever is uncommon in this population contributing to delays in diagnosis which is a risk factor for poor outcome [16]. In a Chinese study comparing CM clinical features between patients with and without predisposing disease, a lower CSF culture positivity rate and longer time to diagnosis was noted in the latter group [17]. Another study identified a non-HIV, non-transplant status as a significant predictor of mortality along with elevated baseline opening pressure and cryptococcemia in a cohort of 302 patients with CM [6].

Cryptococcosis is usually acquired after inhalation of the organism from the environment. In the lungs, it can cause pneumonia. An immunocompetent host will often clear the infection though latent asymptomatic infection can occur in some individuals. Immunosuppression later in life can then lead to reactivation of the infection and dissemination with frequent CNS involvement. Whether reactivation plays more of a causal role in cryptococcosis rather than de novo infection remains unclear [18].

Notably, CM may cause markedly elevated levels of protein and inflammatory material in the CSF that may obstruct flow through neurosurgical devices. A retrospective case review of 50 patients (49/50 with HIV) demonstrated 16 patients required serial LPs and 13 required shunt placement for management of persistent intracranial hypertension with a mean CSF protein level of 88.3 and 59.6, respectively [19]. In a study of tuberculous meningitis managed with ventriculoperitoneal shunts (VPS) in South Africa, it was found that elevated CSF protein levels were associated with shunt obstruction with a mean of 194 mg/dL in the obstructed group as compared to 176 mg/dL in the non-obstructed group [20]. In the present report, persistent elevation of CSF protein and pleocytosis as a result of neuro-inflammation were most probably contributing to recurrent symptoms when multiple attempts to clamp external ventricular drains failed. His presentation was most consistent with PIIRS, a recently described phenomenon in apparently immunocompetent hosts who experience clinical deterioration despite clearing CSF fungal cultures [3, 14]. A predominance of CSF lymphocytes is typical for CM and activated lymphocytes for PIIRS, particularly [3]. This paradoxical immune response is characterized by robust TH1 cell responses in the CSF, with CD4 and CD8 cells activation, increase in inflammatory cytokines, notably IL-6 and soluble CD25 (IL-2 receptor), and a relative decrease in TH2 cell response with a decrease in cytokines such as IL-4 and IL-3 in the setting of CSF fungal culture conversion to negative [3]. Our patient showed clinical improvement with high-dose steroids without conversion of cultures, which points towards a paradoxical immune response. The response in reducing protein and inflammatory debris allowing ventricular pressure control and eventual internalization of the ventricular shunt in this very difficult case points to the facility of corticosteroid therapy in this disease. Despite drainage by EVDs, the persistent entrapment of the occipital and temporal horns did not improve until there was a reduction in choroid plexitis (decreased choroid plexus size and enhancement bilaterally compared to baseline) with corticosteroids, which eventually resulted in improved drainage of the entrapped horns, obviating the need for additional shunt placement within the entrapped ventricular compartments. In previous reports, corticosteroids were given to 8 patients after CSF drainage, which resulted in a decreased intracranial pressure and neurological recovery, with no recurrence of infection, whereas the present case shows the ability of corticosteroids to facilitate internalization of the ventricular catheter itself [21].

In terms of immune susceptibility to *Cryptococcus* in the previously healthy, auto-antibodies have been recognized to play an important role. Neutralizing GM-CSF antibodies are known to predispose to autoimmune pulmonary alveolar proteinosis due to defective surfactant clearance by alveolar macrophages [22], but also have an association with cryptococcal infection in apparently immunocompetent patients, mostly commonly with *C. gattii* rather than *neoformans* [23, 24]. Laboratory studies can help determine the functionality of these antibodies by testing the ability of these antibodies to activate multiple downstream inflammatory signaling pathways including STAT 5, MAP, P13 kinase and NF-κB [13]. These patients generally have a good prognosis with recommended antifungal therapy and a low risk of recurrence [25]. In our patient, an auto-antibody panel performed at the NIH returned positive for anti-GM-CSF antibodies but on further testing for neutralizing activity, did not block the phosphorylation of STAT 5, suggesting an absence of functionality.

The presence of CMV viremia (and in the bone marrow biopsy) in our patient warranted treatment given the continued requirement for high dose corticosteroids. While CMV can cause relative immunosuppression, in our patient, this was most likely reactivation due to corticosteroids and critical illness rather than the primary diagnosis. Thrombocytopenia developed after admission and evaluations did not demonstrate other end-organ involvement that could be attributed to CMV. The choroid plexitis on neuro-imaging is also notable and queries the contribution of CMV to this feature. In a prior study, it was reported that choroid plexitis and ependymitis were exclusively found in HIV-negative CM cases when compared to HIV CM [26]. Therefore, CM and subsequent PIIRS was likely the primary cause of the profound neuroinflammation in this present case. In summary, a predisposing cause or acquired immunodeficiency was not clearly identified for our patient.

The case presentation highlights the consideration of cryptococcal meningitis in the differential diagnosis for neurological symptoms without fever in apparently immunocompetent hosts. Mortality tends to be high, usually due to a delay in diagnosis and robust secondary immune responses. In this case, CSF obstructive phenomena were particularly acute resulting in frequent obstruction of drainage devices and an inability to internalize a ventricular shunt. Thus, high dose corticosteroid therapy in patients with cryptococcal PIIRS may help to facilitate shunt placement and together improve neurological outcomes in such patients.

## Data Availability

Data available from the corresponding author on reasonable request.
